# A 23-gene prognostic classifier for prediction of recurrence and survival for Asian breast cancer patients

**DOI:** 10.1042/BSR20202794

**Published:** 2020-12-02

**Authors:** Ting-Hao Chen, Jian-Ying Chiu, Kuan-Hui Shih

**Affiliations:** Department of Medical Operation, Amwise Diagnostics Pte. Ltd., Singapore

**Keywords:** Asian, classifier model, gene expression profile, recurrence

## Abstract

We report a 23- gene-classifier profiled from Asian women, with the primary purpose of assessing its clinical utility towards improved risk stratification for relapse for breast cancer patients from Asian cohorts within 10 years’ following mastectomy. Four hundred and twenty-two breast cancer patients underwent mastectomy and were used to train the classifier on a logistic regression model. A subset of 197 patients were chosen to be entered into the follow-up studies post mastectomy who were examined to determine the patterns of recurrence and survival analysis based on gene expression of the gene classifier, age at diagnosis, tumor stage and lymph node status, over a 5 and 10 years follow-up period. Metastasis to lymph node (N2-N3) with N0 as the reference (N2 vs. N0 hazard ratio: 2.02 (1.05–8.70), N3 vs. N0 hazard ratio: 4.32 (1.41–13.22) for 5 years) and gene expression of the 23-gene panel (*P*=0.06, 5 years and 0.02, 10 years, log-rank test) were found to have significant discriminatory effects on the risk of relapse (HR (95%CI):2.50 (0.95–6.50)). Furthermore, survival curves for subgroup analysis with N0-N1 and T1-T2 predicted patients with higher risk scores. The study provides robust evidence of the effectiveness of the 23-gene-classifier and could be used to determine the risk of relapse event (locoregional and distant recurrence) in Asian patients, leading to a meaningful reduction in chemotherapy recommendations.

## Introduction

Breast cancer is the second leading cause of cancer deaths in women, next only to lung cancer resulting in 1 death among 38 women each year (6.8%) [1]. Breast cancer remains a very heterogeneous disease comprising a wide range of morphologic and biological features, clinical behavior, and treatment responses [2]. A patient’s prognosis is often judged by the available survival and recurrence rates, usually calculated from diagnosis. The risk of breast cancer recurrence, both local or distant. Is an important criterion that decides treatment courses for patients? Twenty to thirty percent of patients with an early breast cancer are susceptible to relapse [3]. However, factors affecting recurrence/relapse, and the identification of genetic and histological factors that might affect subgroups of patients who continue to be at an increased risk of recurrence long after completing the standard course of treatment, during the first decade following diagnosis, are still an unmet clinical issue.

The risk of recurrence and outcomes have traditionally been stratified with regard to tumor subtype [4,5]. Other characteristic such as tumor size, tumor grade, nodal status, and age of diagnosis affect the disease progression [6]. There is also the hereditary predisposition to breast cancer that accounts for approximately 5–10% of all breast cancers [7]. Breast cancer susceptible gene mutations and the accurate estimation of their probabilities provide vital information towards genetic counselling of breast cancer patients [8]. Multiple factors, such as socioeconomic, epidemiological and genetic etiology, play roles in tumor behavior, cancer subtype and the prognosis of patients, and have been observed to vary among different racial/ethnic groups [9,10]. Mortality rates due to breast cancer also vary across patients from different ancestry. Prior studies have shown that there exist different manifestations of breast cancer risk and prognosis between non-Asian and Asian Triple-negative Breast Cancer (TNBC) subtypes [11]. Such racial differences could be attributed to fundamental epidemiological and genetic risk factors between populations which might be responsible for the underlying mechanism leading to population-specific risk levels. Taking into consideration, effects of ancestry differences, could provide additional understanding of patient prognosis thereby leading to better and appropriate treatment decisions. Therefore, this provides an excellent rationale to conduct breast cancer studies for women specifically from Asian cohorts.

Multiple genetic association studies have accrued over the years and have added to gene-based knowledge for breast cancer. High-penetrance breast cancer susceptibility genes, such as *BRCA1* and *BRCA2*, explain only a small fraction of breast cancers in the general population because of their low carrier rates [12]. Most of the identified genetic factors are associated with only a small to moderate increased risk of breast cancer and therefore, cumulatively may explain a small proportion of heritability of the breast cancer risk. Over the years, several assays have been developed to conduct breast cancer recurrence score tests [13–17]. They provide an indication of the activity of a set of candidate breast cancer-specific genes to predict the probability of the tumor’s growth and spread, thereby act as biomarkers in making treatment decisions following surgery. This is particularly important if the patient requires chemotherapy to prevent the patient from being over treated or undertreated. Oncotype DX [14], Endopredict [13], and RecurIndex [15–17] are such genomic tests that utilize potential breast cancer genetic predictors suitable for patients that are recently diagnosed with early stage, estrogen receptor-positive (ER+) or progesterone receptor-positive (PR+), and Human epidermal growth factor receptor 2-negative (HER2-) breast cancer. These tests typically help make treatment decisions using multigene expression profiles to predict breast cancer’s recurrence or distant metastasis within a follow-up period (maximum 10 years) after diagnosis. Such tests have been effective in preventing overtreatment in early breast cancer patients.

We have reported one 34-gene set and another 18-gene classifier in our prior studies that could partition the loco-regional recurrence in high risk patients from that of the low risk patients after mastectomy [15–17]. Other than gene expression profiling of tumors for predicting clinical outcomes in breast cancer patients, regional lymph node status, and tumor pathological staging may provide surrogate information for events such as metastasis or relapse [18]. Racial and ethnic disparities are evident in underlying genetic and biological factors that might influence the disease incidence and prognosis. Hence, in the present study, we report a 23-gene classifier with the primary purpose of assessing its clinical utility toward improved risk stratification for relapse post mastectomy. The 23 genes are from our published studies, which the metagene we found in the Asian population for the prediction of loco-regional recurrence [15]. After that, we developed and validated the gene-subset from the metagene we found for the prediction of loco-regional recurrence and distant metastasis [16,17]. Based on them, we develop and validate the new subset to predict the relapse of breast cancer in the Asian population. The gene set is derived from genomic profiling of Asian women, to predict the risk level (high/low) of relapse within up to 10 years post mastectomy following initial diagnosis. The present study further establishes the efficacy of the discriminatory 23-genes along with pathological indicators such as tumor stage (T stage) and lymph node status (N stage) to predict survival [15].

## Methods

### Study population

For this Asian population study, a total of 422 patients’ gene expression data were obtained from publicly available gene expression omnibus (GEO) datasets. The first dataset GSE20685 [19] consists of gene expression profiles from 312 prospectively enrolled patients diagnosed with breast cancer and treated between 1991 and 2004 at the Koo Foundation Sun-Yat-Sen Cancer Center (KFSYSCC) and an additional 15 lobular breast carcinoma samples, collected between 1999 and 2004. The second dataset GSE45255 [20] consists of 1954 annotated breast tumors with corresponding clinical–pathological data including distance metastasis-free survival gathered from Singapore and Europe, out of which 95 samples from Singapore origin are included in the present study. Characteristics such as age at diagnosis (years), tumor stage (T1 (stage1), T2 (stage2), T3 (stage3), T4 (stage4)), N stage (lymph nodes status: N0, N1, N2, N3), for each of the samples were recorded. Treatment related status (neo-adjuvant chemotherapy), were also obtained. All women in the present study are treated with either breast-conserving therapy or mastectomy. Patients were classified into different tumor and lymph node and eligible patients met the following inclusion criteria: (1) invasive carcinoma of the breast, (2) clinical stages T1-T4, (3) Lymph node status N0–N3 [19,20]. In addition, 197 out of the 422 patients were entered into the follow-up studies. Remaining patients were excluded if they had (1) no relapse, (2) metastasis at the time of diagnosis (*de novo* stage IV), (3) metastasis after the 10-year period (censored). Data on 197 patients were examined to determine the patterns of recurrences and survival analysis over a 5- and 10-year follow-up period.

### Statistical analysis

**Figure 1:**
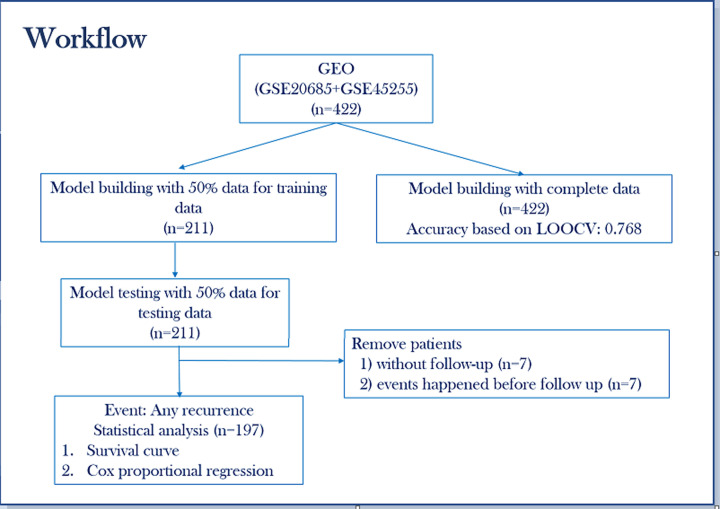
Overview of the workflow for training and testing the 23-gene classifier to predict risk of recurrence and survival analysis, for breast cancer patients from Asian cohorts

Figure 1 summarizes the workflow that is implemented in the present study. A previously identified 23 prognosis-related genes classifier is utilized to predict overall recurrence based on their binary expression status.

### Model building

A logistic model was built using all study samples and a leave one out cross-validation (LOOCV) [21] procedure is conducted to check the accuracy and test the preliminary performance of 23-gene set. LOOCV provides an almost unbiased estimate of generalization performance [22] and consists of training the model on *n* - 1 subsamples and the model selection criterion is evaluated on the remaining 1 sample. This procedure is then repeated for all *n* combinations of n - 1 subsamples and subsequently the accuracy is calculated to judge the model performance.

### Model training and testing

Logistic regression was used to predict the recurrence of breast cancer based on the 20 gene expression as the predictor. The model is built in R where the outcome variable is binomial with a link function of logit. The binary response parameter is recurrence (*y* = 1) or disease-free (*y* = 0). Selection of a best fit logistic regression model is accomplished through model training and leads to obtaining optimal values of prediction-parameters by which the model is governed. The model is trained using a supervised learning method. The predicted y (recurrence/disease-free) for the model is run with 50% of the total samples as training samples, and the predicted (*y*) value (predicted high or predicted low risk) is then compared with the respective observed status (observed relapse or disease-free) of each patient, using the input vector of *x’*s (gene expression of 23 genes) as the predictor. Based on the result of the comparison and the specific learning algorithm being used, the parameters of the model are adjusted.

Once the model is trained, the model is tested to determine how accurately the predictive model will perform in practice. The remaining 50% of the total samples are used as the test dataset to provide an unbiased evaluation of a final model that is fit on the training dataset. The model performance is evaluated through the confusion matrix while the clinical performance is judged through metrics such as sensitivity, specificity, positive predictive value (PPV) and negative predictive value (NPV). The clinical performance from the model and confusion matrix were presented through R package “DescTools”(https://cran.r-project.org/web/packages/DescTools/index.html). All of the model training and testing are conducted on the R version 3.6.3.

### Recurrence analysis

We previously reported a 23-gene classifier which includes *ACTB, BLM, BUB1B, CCR1, CKAP5, CLCA2, DDX39, DTX2, ERBB2, ESR1, MKI67, OBSL1, PGR, PHACTR2, PIM1, PTI1, RCHY1, RPLPO, SF3B5, STIL, TFRC, TPX2*, and *YWHAB* along with 3 housekeeping genes *ACTB, RPLP0, and TFRC* (not included in this study). These genes are selected through our previous studies [15–17]. Quantile normalization was used to normalized the raw data using the R package ‘preprocessCore’. (https://github.com/bmbolstad/preprocessCore). For survival analysis (1) subjects were removed without follow-up data, (2) subjects with recurrence, before the surgery date, were excluded, and (3) subjects who recorded recurrence after 10 years from initial diagnosis were censored and were not included in the analysis.

### Survival analysis

Cox proportional hazards regression models were used to assess the prognostic significance of age at diagnosis, pathological tumor grade, N-stage, and the 23-gene classifier. R packages survminer (https://cran.r-project.org/web/packages/survminer/index.html) and survival (https://cran.r-project.org/web/packages/survival/survival.pdf) were used to conduct all survival analysis. Disease-free interval is displayed by Kaplan–Meier method and log-rank test is used to determine any statistically significant differences in survival between the indicated groups. Comparative analyses were performed between groups using Chi-squared and T-tests for categorical and numeric variables. Statistical significance was accepted for *P* < 0.05. Both univariate and multivariable Cox proportional hazard analyses were performed for age, T and N staging, and gene expression profiles, for both 5- and 10-year follow-up data to obtain hazard ratios (HRs) with 95% confidence intervals (CIs) and *P*-values. Finally, a subgroup analysis using Cox-proportional hazard test stratified by tumor stage T1-T2 and N-stage N0-N1, respectively, were conducted to estimate if they had any significant effect in predicting the survival of patients within a 10-year follow-up period from the initial diagnosis.

## Results

### Patient demographics

About 327 patients from Gene expression Omnibus (GEO)) dataset GSE20685 [19], who underwent modified radical mastectomy or breast-conserving surgery plus dissection of axillary nodes were included in the present study. Clinical information on the follow up treatments for these patients included radiotherapy for 141 patients, adjuvant chemotherapy for 232 patients, and/or hormonal therapy for 224 patients. Neoadjuvant chemotherapy was administered to 31 patients with locally advanced disease. Similar clinical information from the 95 patients from the Singapore cohort (GSE45255) [20] were presented with tamoxifen monotherapy (ER+), chemotherapy, and/or neoadjuvant chemotherapy status. The demographic features for the 422 (total) patients, included in the present study, such as age at diagnosis, N stage (N0,N1,N2,N3), tumor stage (T1, T2, T3, T4), recurrence (relapse or disease-free) and follow-up status are summarized in Table 1. To further determine the recurrence and survival rate of the patients, further 5 and 10 years follow-up studies were conducted on a total of 197 patients, because there were 7 patients without follow-up and 7 events happened before follow up. The detail is displayed on Figure 1. The demographic details of the follow-up patient sample with age at diagnosis, tumor stage, N stage, and recurrence status are displayed in Table 2.

**Table 1:** Demography of the overall samples included diagnosed with breast cancer

Overall study samples	
***n***	422
Age (mean(SD))	49.17 (11.26)
N Stage (%)	
0	187 (44.3)
1	132 (31.3)
2	63 (14.9)
3	40 (9.5)
Tumor stage (%)	
1	126 (29.9)
2	252 (59.7)
3	32 (7.6)
4	12 (2.8)
Any Recurrence = Yes (%)	103 (24.4)
DFI follow-up (median [IQR])	6.45 [4.33, 9.60]

**Table 2:** Demographic table for classification by prediction model for 5- and 10-year follow-up data

Terms	Model prediction		*P*-value
	High-risk	Low-risk	
*N*	19	178	
Age (mean (SD))	49.00 (9.72)	49.99 (11.33)	0.713
*N* stage (%)			0.979
0	9 (47.4)	87 (48.9)	
1	6 (31.6)	53 (29.8)	
2	2 (10.5)	23 (12.9)	
3	2 (10.5)	15 (8.4)	
Tumor stage (%)			0.567
1	6 (31.6)	60 (33.7)	
2	10 (52.6)	101 (56.7)	
3	3 (15.8)	13 (7.3)	
4	0 (0.0)	4 (2.2)	
Any recurrence = Yes (%)			
5-year follow-up	5 (29.4)	24 (14.1)	0.190
10-year follow-up	7 (36.8)	31 (17.5)	0.085
DFI follow-up (median [IQR])			
5-year follow-up	5.00 [1.25,5.00]	5.00[4.65,5.00]	0.183
10-year follow-up	5.88[1.25,8.85]	6.45[4.65,9.47]	0.282

### Training and testing performance of the 23 gene classifier

**Figure 2:**
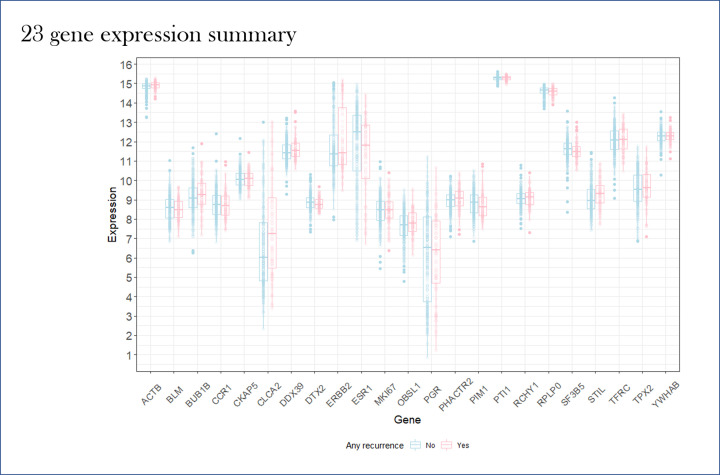
Gene expression profiles for each of the genes in the 23-gene panel for 422 breast cancer patients with and without recurrence respectively *X*-axis: Gene names of 23 genes from the gene-classifier; *Y-*axis: gene expression values; pink box plots: recurrence; blue box plots: no recurrence.

Gene expression profiles for each of the genes in the 23-gene panel is displayed in Figure 2 for all patients with and without recurrence respectively. The gene expression profile displayed all genes with high median gene expression (log2 expression >7) for both recurrence free patients as well as for patients with recurrent breast cancer. The expression profiles pointed out that ACTB, *PTI1*, and *RPLPO* to be consistently high across patients with and without recurrence.

The predictive power of the gene classifier is established through accuracy, sensitivity, specificity, PPV, and NPV measures for the fitted logistic regression model for patients at high risk versus low risk of recurrence. Table 3A,B summarizes the confusion matrix for predicted and observed recurrence risks (high/low) in patients from both training and testing data. While the model achieved a training accuracy of 78.7% (Table 3A), it achieved a testing accuracy of 73.9% (Table 3B). The ability of the model to correctly classify a high-risk patient was 23.6% (training sensitivity) and 15.7% (testing sensitivity); however, the probability of correctly classifying a low risk individual correctly (specificity) was 96.9% (training) and 92.5% (testing). Further, the PPV and NPV of the classifier reached 70.6% and 79.4% for the training data whereas it could just achieve a PPV of 40% and NPV of 77.5% for the testing data.

**Table 3:** Demographic table for classification by prediction model

(A)			(B)		
Confusion Matrix Model: Gentic (training data)			Confusion Matrix Model: Gentic (testing data)		
Pred/Obs	Yes	No	Pred/Obs	Yes	No
**High-risk**	12	5	**High-risk**	8	12
**Low-risk**	40	154	**Low-risk**	43	148
**Accuracy**	0.787		**Accuracy**	0.739	
**Sensitivty**	0.231		**Sen**	0.157	
**Specificity**	0.969		**Spec**	0.925	
**PPV**	0.706		**0PPV**	0.400	
**NPV**	0.794		**NPV**	0.775	

### 5- and 10-year follow-up analysis

**Figure 3:**
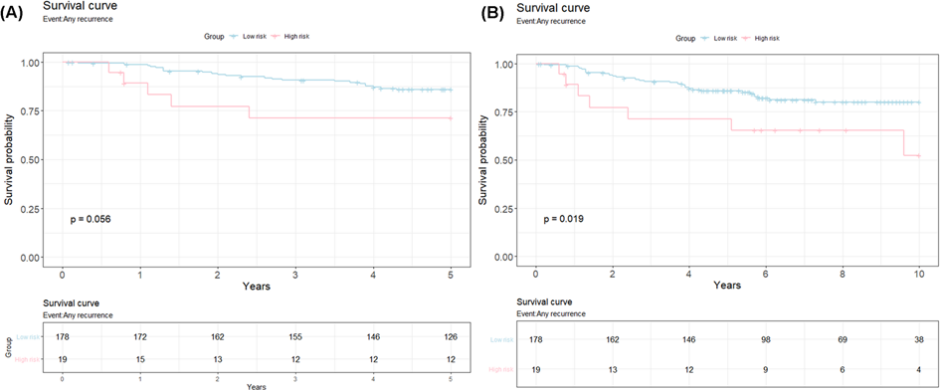
The survival curves from the Cox-Proportional Regression models for patients with high-risk versus low risk for relapse (**A**) Survival curve for 5-year follow-up study; *X*-axis- years till event; *Y*-axis-Survival probability; Pink: High risk; Blue: low risk; (**B**) Survival curve for 10-year follow-up study; *X*-axis- years till event; *Y*-axis-Survival probability; Pink: High risk; Blue: low risk.

Table 2 summarizes the demographic table for classification by the prediction model for a 5-year (median follow-up: 5.00 [4.29–5.00]) and 10-year recurrence (median follow-up: 6.28 [4.29–9.4]), respectively. 197 patients were retained for the 5/10-year follow-up study of which 19 were predicted to be at high risk of recurrence, with a mean age of 49 years, of which 5 (29.4%) relapsed within 5 years and 7 (36.8%) relapsed within 10 years, and 178 as low risk to recurrence with a mean age of 50 years of which 24 (14%) relapsed in 5 years and 31 (17.4%) in 10 years. The performance of risk prediction for patients separated by lymph node status (N stages: N0 – N3) and tumor stages (T1 – T4) are displayed with *P*-values 0.97 and 0.56, respectively, for 5 and 10 years. Figure 3 shows the survival curves for patients with high-risk versus low risk for relapse in patients up to 5 and 10 years, from date of diagnosis. The survival analysis predicted the relapse rate to be 0.71 (confidence interval (95%CI):0.53–0.96) (up to 5 years) and 0.52 (95%CI: 0.3–0.91) (up to 10 years) for high risk patients and 0.86 (95%CI: 0.81–0.91) (up to 5 years) and 0.8 (95%CI*:*0.74–0.87) (up to 10 years) for low-risk patients with a *P*-value of 0.056 and 0.019, respectively. This indicates that patients with high risk scores displayed high relapse rates than those with low risk scores and there was significant difference between relapse free between high and low risk groups. To delve deeper into the intricacies of how the relapse is affected by each of the factors (genetic, age at diagnosis, T and N stage) univariate and multivariate Cox proportional hazard test results were studied. The analysis results for univariate Cox proportional hazard test for effects of covariates on patient prognosis between high risk and low risk groups, shows that difference in risk to relapse is not attributed to age at diagnosis and tumor stage (Table 4). However, metastasis to lymph node (N2 and N3) with N0 as the reference (*P*=0.01 (5 years), 0.001 (10 years)) and gene expression of the 23-gene panel (*P*=0.06 (5 years), 0.02 (10 years)) were found to have a discriminatory effect on the risk of relapse (HRs (95%CI) N2 vs. N0: 2.02 (1.05–8.70), N3 vs. N0: 4.32 (1.41–13.22) for 5 years). Next, multivariate Cox proportional regression analysis using all the factors (age at diagnosis, N stage, tumor stage and gene expression) as predictors for survival (5 and 10 years) between high risk versus low risk patients confirmed the findings from univariate analysis with hazard ratios ≥2 and *P*<0.05 for gene expression (HRs (95%CI), for 5 years follow: 2.50 (0.95–6.59) and for 10 years follow: 2.65 (1.16–6.08)) (Table 4).

**Table 4:** Cox-proportion regression tests on 5- and 10-year follow-up data

Term	Univariate Cox proportional regression		Multiple Cox proportional regression	
	HR (95% CI for HR)	*P*-value	HR (95% CI for HR)	*P*-value
(A)				
Age	0.99 (0.96-1.02)	0.536	0.99 (0.96-1.03)	0.735
N Stage				
0	(reference)		(reference)	
1	2.07 (0.82-5.24)	0.126	2.05 (0.80-5.22)	0.134
2	2.02 (1.05-8.70)	0.041	3.08 (1.02-9.28)	0.045
3	4.32 (1.41-13.22)	0.010	4.13 (1.32-12.93)	0.015
Tumor Stage				
T1	(reference)		(reference)	
T2-T4	1.36 (0.60-3.07)	0.461	1.03 (0.44-2.41)	0.951
Genetic				
Low	(reference)		(reference)	
High	2.48 (0.95-6.50)	0.065	2.50 (0.95-6.59)	0.063
(B)				
Age	0.99 (0.97-1.02)	0.723	0.99 (0.97-1.03)	0.935
N Stage				
0	(reference)		(reference)	
1	2.21 (0.97-5.04)	0.059	2.20 (0.96-5.06)	0.062
2	3.11 (1.23-7.89)	0.017	3.54 (1.34-9.36)	0.011
3	4.84 (1.84-12.72)	0.001	4.99 (1.86-13.46)	0.001
Tumor Stage				
T1	(reference)		(reference)	
T2-T4	1.13 (0.57-2.24)	0.723	0.83 (0.40-1.71)	0.615
Genetic				
Low	(reference)		(reference)	
High	2.58 (1.13-5.85)	0.024	2.65 (1.16-6.08)	0.021

### Subgroup analysis

**Figure 4:**
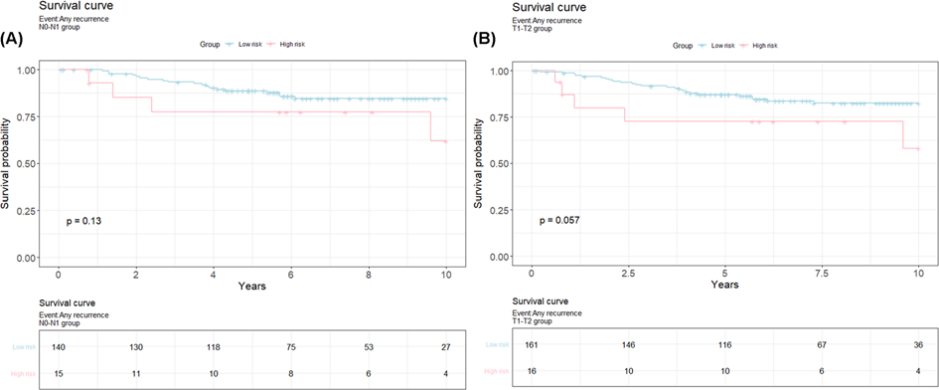
Survival curves for the subgroup analysis from the Cox-proportional regression models for patients with high-risk versus low risk for relapse (**A**) Survival curve for N0-N1 subgroup for a 10-year follow-up study; *X*-axis- years till event; *Y*-axis-Survival probability; Pink: High risk; Blue: low risk. (**B**) Survival curve for T1-T2 subgroup for a 10-year follow-up study; *X*-axis- years till event; *Y*-axis-Survival probability; Pink: High risk; Blue: low risk.

To evaluate the 23-gene classifier, based on its ability to predict recurrence in early stage patients’, tumor grades 1 and 2 and N-stage 0 and 1 were chosen for a further subgroup analysis. Moreover, the findings from the Cox proportional hazard analysis in the previous step, along with the fact that the most frequent groups of patients in a 10-year follow-up study were with T stage 1 and 2 (89% of the total patients in follow up study) and N Stage 0 and 1 (78% of the total follow up patients, Table 2), led us to focus on the mentioned subgroups. Also, a larger tumor burden and higher metastatic risk restricts the benefit the classifier may provide, while a lower tumor burden in N1 might help to improve survival. Figure 4 displays the survival curves for the subgroup analysis, obtained through Kaplan Meier analysis, which showed that patients with high risk scores reported shorter survival rates compared with those with low risk scores. There was no significant difference in overall survival between high and low risk patients according to N stage (N0-N1) (*P*=0.13) and marginally significant discriminatory effect of tumor stage (T1-T2) (*P*=0.057) between high risk versus low risk.

## Discussion

A promising new strategy that is utilized avidly for predicting clinical outcomes in breast cancer patients is gene expression profiling of tumors. Some studies in the past have established that the clinical responses for patients are correlated with different specific molecular ‘portraits’ [23] and gene signatures can potentially distinguish subgroups of patients with different prognoses or response to different treatment regimens [24]. In the present study we, therefore, try to establish the effectiveness of a gene classifier as a predictor for recurrence, post adjuvant chemotherapy, in breast cancer diagnosed patients from Asian population. The gene-classifier consists of a 23 gene cluster of correlated genes that is used to predict patient risk stratification (high risk or low risk) for relapse within 10 years from diagnosis where the model parameters are first trained to obtain the best fit model. Once a best fit model is obtained it is tested on a separate dataset, to evaluate the feasibility of the model. The classifier is designed to be used for decision making through a logistic regression model. It is a major challenge to validate gene classifiers with samples that are independent from those that were used to develop them [25]. Increased sensitivity, the ability to correctly identify people with high risk of recurrence, usually comes at the expense of reduced specificity (meaning more false-positives). Likewise, high specificity, when a test does a good job of ruling out people with low risk of recurrence, usually means that the test has lower sensitivity (more false-negatives). A false negative result might be giving false reassurance to the patient and a false-positive result might send patients on unnecessary and expensive medical procedures. Therefore, despite good accuracy, low sensitivity, or high specificity and high PPV and NPV, careful consideration of interplay of diagnostic accuracy is vital for reaching a reliable conclusion.

It is not enough to make decisions based only on gene expression as the complexity of a classification can be affected by so many other factors that drive the prognosis of a complex disease such as breast cancer. Other criteria such as tumor size, nodal status, age at diagnosis along with genetic etiology affects the diseases prognosis, relapse/disease-free and survival. We conducted a 5- and 10-year follow‐up, taking into consideration the role of each prognostic factor in predicting the course of the disease and the response to different treatment strategies. Differences in recurrence risks between prognostic subgroups was observed to decline over time. Also differences in disease-free survival between subgroups decreased with time as well. All results indicate that conditional survival and recurrences stratified by gene-panel and lymph node status provide patients better insight in prognosis. The significant *P*-values and overall hazard ratios also confirmed the success of the gene classifier to discriminate patients with high risk from patients with low risk to metastasis.

The 23-gene classifier, of which three were housekeeping genes and were not included, conform mostly to subsets of patients of Asian origin. Pooling all of the expression of the genes in the classifier, *ACTB, BLM, BUB1B, CCR1, CKAP5, CLCA2, DDX39, DTX2, ERBB2, ESR1, MKI67, OBSL1, PGR, PHACTR2, PIM1, PTI1, RCHY1, RPLPO, SF3B5, STIL, TFRC, TPX2*, and *YWHAB* indicates an increased likelihood of breast cancer [26–28]; however, membership in a prognostic gene list is not necessarily indicative of a gene’s importance in cancer pathology. Nevertheless, each of the genes listed has been associated with some or more cancer related prognosis. The scoring algorithm was formulated by assigning each gene with a weigh according to the logistic regression before assembling the scores to one gene score that was used as a cut-off to classify patients. PHACTR2 is associated with up-regulated and down-regulated functional gene networks of the migratory breast tumor cells [29], TPX2 suppresses activation of p53 pathway in breast cancer [30], DDX39 involved in embryogenesis, spermatogenesis, and cellular growth and division. The following genes maybe have protective effect for the prognosis of breast cancer, BLM has a key role in homologous recombination repair, telomere maintenance, and DNA replication [28]. The molecular function of SF3B5 is about RNA binding and Splicing factor binding. The gene panel is consistent with our earlier study [15]. Moreover, extracting biological meaning from whole genome molecular profiling remains a significant challenge.

There have also been concerns relating to design and validation of gene classifiers, as small numbers of patient samples are usually used to derive classifiers and there is very little overlap present in these gene signatures among various classifiers. The Oncotype DX Breast Cancer Assay, developed by Genomic Health, is a 21-gene assay that can predict disease recurrence and response to chemotherapy in ER positive, HER2-negative, early stage breast cancer. EndoPredict combines prognostic information from a 8-gene analysis with tumor size and the patient's nodal status. Both tests have been developed to guide treatment decisions on whether women should receive chemotherapy in addition to anti-hormone treatments. A prior study has reported that the 8-gene signature obtained from the EndoPredict breast cancer test might be more accurate at predicting the recurrence of breast cancer, compared with the recurrence score of Oncotype DX [31]. The 23-gene set was selected from and validated on several datasets and was inferred as the most stable gene-set to predict the relapse of breast cancer. The performance of the gene classifier in this study is very encouraging, as the survival benefit was more significant in follow-up and subgroup analysis. However, comparison of our predictor with other predictors, needs to be evaluated as patients who are correctly predicted to be at low risk can be saved the unwanted side effects that are common with chemotherapy treatments and can have better life quality.

Gene expression profiling of tumors appears to be a promising new strategy for predicting clinical outcomes in breast cancer patients.

## Conclusion

The present study aims to determine the risk of recurrence in breast cancer patients from Asia by utilizing a 23 gene prognostic signature. It is designed to validate the gene signature through a 10-year follow-up study (recurrence and overall survival) and further define the prognosis of breast cancer and assessment of patients where chemotherapy will be beneficial. The study provides robust evidence of the clinical utility of the 23-gene classifier and that it can successfully distinguish between patients with high and low risk of relapse. Such information are potential molecular tools for clinicians to help them with selection of therapeutic strategies, such as extension of adjuvant endocrine therapy or on suppressing adjuvant chemotherapy in patients were toxic effects are particularly deleterious or when this treatment is fundamentally not needed.

## Data Availability

The data used to support this study was in the manuscript. The dataset is available in NCBI GEO database, which with the accession number GSE20685 and GSE45255.

## Abbreviations

CI: confidence interval
ER: estrogen receptor
HER: human epidermal growth factor receptor
HR: hazard ratio
PPV: positive predictive value
PR: progesterone receptor

## References

[1] Nkondjock A. and Ghadirian P. (2005) Risk factors and risk reduction of breast cancer. Med. Sci. 21, 175–180 10.1051/medsci/2005212175 15691489

[2] Turashvili G. and Brogi E. (2017) Tumor heterogeneity in breast cancer. Front. Med. 4, 227 10.3389/fmed.2017.00227 PMC5727049 29276709

[3] Sánchez C. et al. (2016) Clinico-pathologic subtypes of breast cancer primary tumors are related to prognosis after recurrence. Asian Pac. J. Cancer Prev. 17, 5081 28122438 10.22034/APJCP.2016.17.12.5081 PMC5454640

[4] Fragomeni S.M., Sciallis A. and Jeruss J.S. (2018) Molecular subtypes and local-regional control of breast cancer. Surg. Oncol. Clin. 27, 95–120 10.1016/j.soc.2017.08.005 PMC5715810 29132568

[5] McAnena P.F. et al. (2018) Breast cancer subtype discordance: impact on post-recurrence survival and potential treatment options. BMC Cancer 18, 203 10.1186/s12885-018-4101-7 29463223 PMC5819681

[6] Carter C.L., Allen C. and Henson D.E. (1989) Relation of tumor size, lymph node status, and survival in 24,740 breast cancer cases. Cancer 63, 181–187 10.1002/1097-0142(19890101)63:1<181::AID-CNCR2820630129>3.0.CO;2-H 2910416

[7] Larsen M.J., Thomassen M., Gerdes A.-M. and Kruse T.A. (2014) Hereditary breast cancer: clinical, pathological and molecular characteristics. Breast Cancer: Basic Clin. Res. 8, S18715, BCBCR 10.4137/BCBCR.S18715 PMC4213954 25368521

[8] Meiser B. et al. (2008) Genetic counselling and testing for inherited gene mutations in newly diagnosed patients with breast cancer: a review of the existing literature and a proposed research agenda. Breast Cancer Res. 10, 216 10.1186/bcr2194 19090970 PMC2656887

[9] Wheeler S.B., Reeder-Hayes K.E. and Carey L.A. (2013) Disparities in breast cancer treatment and outcomes: biological, social, and health system determinants and opportunities for research. Oncologist 18, 986 10.1634/theoncologist.2013-0243 23939284 PMC3780646

[10] Momenimovahed Z. and Salehiniya H. (2019) Epidemiological characteristics of and risk factors for breast cancer in the world. Breast Cancer: Targets Ther. 11, 151 10.2147/BCTT.S176070 PMC6462164 31040712

[11] Tseng L.-M et al. (2017) A comparison of the molecular subtypes of triple-negative breast cancer among non-Asian and Taiwanese women. Breast Cancer Res. Treat. 163, 241–254 10.1007/s10549-017-4195-7 28299476 PMC5410215

[12] Zheng W. et al. (2010) Genetic and clinical predictors for breast cancer risk assessment and stratification among Chinese women. J. Natl. Cancer Inst. 102, 972–981 10.1093/jnci/djq170 20484103 PMC2897876

[13] Sestak I. et al. (2019) Prediction of chemotherapy benefit by EndoPredict in patients with breast cancer who received adjuvant endocrine therapy plus chemotherapy or endocrine therapy alone. Breast Cancer Res. Treat. 176, 377–386 10.1007/s10549-019-05226-8 31041683 PMC6555778

[14] Schildgen V., Warm M., Brockmann M. and Schildgen O. (2019) Oncotype DX Breast Cancer recurrence score resists inter-assay reproducibility with RT 2-Profiler Multiplex RT-PCR. Sci. Rep. 9, 1–14 10.1038/s41598-019-56910-0 31889145 PMC6937305

[15] Cheng S.H. et al. (2006) Genomic prediction of locoregional recurrence after mastectomy in breast cancer. J. Clin. Oncol. 24, 4594–4602 10.1200/JCO.2005.02.5676 17008701

[16] Cheng S.H. et al. (2016) An eighteen-gene classifier predicts locoregional recurrence in post-mastectomy breast cancer patients. EBioMedicine 5, 74–81 10.1016/j.ebiom.2016.02.022 27077114 PMC4816846

[17] Cheng S.H.-C. et al. (2017) Validation of the 18-gene classifier as a prognostic biomarker of distant metastasis in breast cancer. PLoS ONE 12, e0184372 10.1371/journal.pone.0184372 28886126 PMC5590926

[18] Lu X. et al. (2008) Predicting features of breast cancer with gene expression patterns. Breast Cancer Res. Treat. 108, 191 10.1007/s10549-007-9596-6 18297396

[19] Kao K.-J., Chang K.-M., Hsu H.-C. and Huang A.T. (2011) Correlation of microarray-based breast cancer molecular subtypes and clinical outcomes: implications for treatment optimization. BMC Cancer 11, 143 10.1186/1471-2407-11-143 21501481 PMC3094326

[20] Nagalla S. et al. (2013) Interactions between immunity, proliferation and molecular subtype in breast cancer prognosis. Genome Biol. 14, R34 10.1186/gb-2013-14-4-r34 23618380 PMC3798758

[21] Stephenson A.J. et al. (2005) Integration of gene expression profiling and clinical variables to predict prostate carcinoma recurrence after radical prostatectomy. Cancer: Interdiscipl. Int. J. Am. Cancer Soc. 104, 290–298 10.1002/cncr.21157 PMC1852494 15948174

[22] Luntz A. and Brailovsky V. (1969) On estimation of characters obtained in statistical procedure of recognition. Technicheskaya Kibernetica

[23] Prat A. and Perou C.M. (2011) Deconstructing the molecular portraits of breast cancer. Mol. Oncol. 5, 5–23 10.1016/j.molonc.2010.11.003 21147047 PMC5528267

[24] Hallett R.M., Dvorkin-Gheva A., Bane A. and Hassell J.A. (2012) A gene signature for predicting outcome in patients with basal-like breast cancer. Sci. Rep. 2, 227 10.1038/srep00227 22355741 PMC3259129

[25] Loi S. et al. (2008) Predicting prognosis using molecular profiling in estrogen receptor-positive breast cancer treated with tamoxifen. BMC Genomics 9, 239 10.1186/1471-2164-9-239 18498629 PMC2423197

[26] Fu X. et al. (2016) Overexpression of BUB1B contributes to progression of prostate cancer and predicts poor outcome in patients with prostate cancer. OncoTargets Ther. 9, 2211 27143916 10.2147/OTT.S101994 PMC4844448

[27] Sassi A., Popielarski M., Synowiec E., Morawiec Z. and Wozniak K. (2013) BLM and RAD51 genes polymorphism and susceptibility to breast cancer. Pathol. Oncol. Res. 19, 451–459 23404160 10.1007/s12253-013-9602-8 PMC3708281

[28] Arora A. et al. (2015) Transcriptomic and protein expression analysis reveals clinicopathological significance of bloom syndrome helicase (BLM) in breast cancer. Mol. Cancer Ther. 14, 1057–1065 10.1158/1535-7163.MCT-14-0939 25673821

[29] Patsialou A., Wang Y., Lin J. et al. (2012) Selective gene-expression profiling of migratory tumor cells in vivo predicts clinical outcome in breast cancer patients. Breast Cancer Res. 14, R139 10.1186/bcr3344 23113900 PMC4053118

[30] Chen M., Zhang H., Zhang G., Zhong A., Ma Q., Kai J. et al. (2018) Targeting TPX2 suppresses proliferation and promotes apoptosis via repression of the PI3k/AKT/P21 signaling pathway and activation of p53 pathway in breast cancer. Biochem. Biophys. Res. Commun. 507, 74–82 10.1016/j.bbrc.2018.10.164 30454896

[31] Buus R., Sestak I., Kronenwett R. et al. (2016) Comparison of EndoPredict and EPclin with Oncotype DX recurrence score for prediction of risk of distant recurrence after endocrine therapy. J. Natl. Cancer Inst. 108, pii: djw149 10.1093/jnci/djw149 PMC5241904 27400969

